# Myeloid C‐type lectin receptors in skin/mucoepithelial diseases and tumors

**DOI:** 10.1002/JLB.2RI0119-031R

**Published:** 2019-04-09

**Authors:** Ce Tang, Yulia Makusheva, Haiyang Sun, Wei Han, Yoichiro Iwakura

**Affiliations:** ^1^ Center for Animal Disease Models Research Institute for Biomedical Sciences Tokyo University of Science Noda Japan

**Keywords:** C‐type lectin receptor, colitis, asthma, psoriasis, atopic dermatitis, cancer, fungal infection, mycobacterium infection, mucosal immunity, innate immunity

## Abstract

Myeloid C‐type lectin receptors (CLRs), which consist of an extracellular carbohydrate recognition domain and intracellular signal transducing motif such as the immunoreceptor tyrosine‐based activation motif (ITAM) or immunoreceptor tyrosine‐based inhibitory motif (ITIM), are innate immune receptors primarily expressed on myeloid lineage cells such as dendritic cells (DCs) and Mϕs. CLRs play important roles in host defense against infection by fungi and bacteria by recognizing specific carbohydrate components of these pathogens. However, these immune receptors also make important contributions to immune homeostasis of mucosa and skin in mammals by recognizing components of microbiota, as well as by recognizing self‐components such as alarmins from dead cells and noncanonical non‐carbohydrate ligands. CLR deficiency not only induces hypersensitivity to infection, but also causes dysregulation of muco‐cutaneous immune homeostasis, resulting in the development of allergy, inflammation, autoimmunity, and tumors. In this review, we introduce recent discoveries regarding the roles of myeloid CLRs in the immune system exposed to the environment, and discuss the roles of these lectin receptors in the development of colitis, asthma, psoriasis, atopic dermatitis, and cancer. Although some CLRs are suggested to be involved in the development of these diseases, the function of CLRs and their ligands still largely remain to be elucidated.

## Graphical abstract

Review of the roles of myeloid C‐type lectin receptors, which regulate immune responses in muco‐epithelium, skin, and tumors, in modulating the development of colitis, asthma, psoriasis, atopic dermatitis, and cancer.

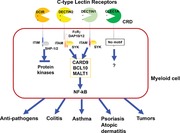

**Abbreviations**
ABPA: allergic bronchopulmonary aspergillosis
AD: atopic dermatitis
AHR: airway hypersensitivity
cDC: conventional DC
CRD: carbohydrate recognition domain
CTL: C‐type lectin
CLR: CTL receptor
DC: dendritic cell
DSS: dextran sulfate sodium
HDM: house dust mite
IBD: inflammatory bowel diseases
ILC3: type 3 innate lymphoid cell
IMQ: imiquimod
ITAM: immunoreceptor tyrosine‐based activation motif
ITIM: immunoreceptor tyrosine‐based inhibitory motif
MDSC: myeloid‐derived suppressor cell
PRR: pattern recognition receptor
ROS: reactive oxygen species
TDM: trehalose 6, 6′‐dimycolate

## 1 INTRODUCTION

C‐type lectins (CTLs) are one of pattern recognition receptors (PRRs) that recognize pathogen‐associated molecular patterns (PAMPs) of pathogens. CTL molecules contain a carbohydrate recognition domain (CRD) in the C‐terminus that recognizes specific carbohydrate structures on pathogens in a Ca^2+^‐dependent manner. Many CTLs also contain signaling motifs, such as immunoreceptor tyrosine‐based activation motif (ITAM) or immunoreceptor tyrosine‐based inhibitory motif (ITIM) in the cytoplasmic portion, or recruit an adaptor protein that contains ITAM, thus acting as a signaling receptor for pathogens (CTL receptor, CLR; Table 1). Some family members can recognize molecules other than carbohydrates in a Ca^2+^‐independent manner. CTLs are consisted of more than 100 family molecules and divided into 16 groups.^1^ Myeloid CLRs belong mainly to groups 2, 5, and 6 of the CTL family. Among them, the genes encoding DCAR2, DCIR, DCAR1, DECTIN‐2, MCL, MINCLE, CLEC12B, CLEC2 DNGR‐1, CLEC1A, DECTIN‐2, and LOX‐1 map to the *Dectin‐1* and *Dectin‐2* cluster loci on mouse chromosome 6 (Table 1) and chromosome 12 in humans. Genes encoding other myeloid CLRs, such as DC‐SIGN (human Chr. 19) and its mouse homologue SIGNR3 (mouse Chr. 8), LANGERIN, MGL, MDL‐1, DCAL‐1, MR, and Dec‐205 map to other chromosomes. These molecules are expressed as membrane proteins in myeloid cells including monocytes, Mϕs, and dendritic cells (DCs) (Table 1).

**Table 1:** Myeloid C‐type lectin receptors in mice

Name	Other names	Gene symbol(Chr. #)	Signaling motif	Ligand	Expression	Function
CLEC1A	C‐type lectin‐like receptor‐1, CLEC1, MelLec	Clec1a (Chr. 6, Dectin‐1 cluster)	No known motif	DHN‐melanin	Endothelial cells, DC	Immune response against *Aspergillus fumigatus*; Allograft tolerance
CLEC2	C‐type lectin‐like receptor‐2, CLEC1B	Clec1b (Chr. 6, Dectin‐1 cluster)	Hemi‐ITAM	Podoplanin, rhodocytin	Platelets, megakaryocytes, Kupfer cells	Lymphvasculogenesis; maintenance of hematopoetic stem cells; regulate tumor cell growth
CLEC12B	C‐type lectin‐like receptor‐12B	Clec12b (Chr. 6, Dectin‐1 cluster)	ITIM	Minor binding to terminal GlcNAc, GalNAc and galactose	In vitro differentiated Mϕ, Caveolin‐1‐dependent expression	Inhibition of the NK receptor NKG2D‐mediated signaling
DCAR1	Mouse dendritic cell immune activating receptor 1, Apra1	Clec4b2 (Chr. 6, Dectin‐2 cluster)	None.	No known ligand	CD8^+^ DC, CD11b^+^ myeloid cells	Enhancement of inflammatory response
DCAR2	Dendritic cell immunoactivating receptor, DCAR, Dcar2, Apira2, DCARbeta	Clec4b1 (Chr. 6, Dectin‐2 cluster)	None. Association with ITAM‐containing FcRg	Phosphatidyl‐inositol mannosides (PIM)	Mϕ, Mo‐derived cells	T cell response against mycobacteria
DCIR	Dendritic cell immune‐receptor, DCIR1	Clec4a2 (Chr. 6, Dectin‐2 cluster)	ITIM	Sulfated lactose, LacNAc, biantennary N‐glycans	DC, Mϕ, Neu, B cells	DC and osteoclast differentiation; immunity to tuberculosis; attachment of HIV and HCV to facilitate infection
DEC‐205	CD205	Ly75 (Chr. 2)	Tyr‐based motif	Keratins	Mature DC, LC, thymic epithelial cells	Endocytosis of Ags; Ag cross presentation; recognition of dead cells
DECTIN‐1	Dendritic cell‐associated C‐type lectin‐1, CLECSF12	Clec7a (Chr. 6, Dectin‐1 cluster)	Hemi‐ITAM (YxxL motif)	b‐glucans, galectin‐9, tumor‐specific carbohydrate	DC, Mϕ, LC	Defense against fungi and mycobacteria; tumor promotion; protection against tumors
DECTIN‐2	Dendritic cell‐associated C‐type lectin‐2, CLEC6A	Clec4n (Chr. 6, Dectin‐2 cluster)	None, association with ITAM‐containing FcRg	α‐mannans, Man‐LAM	DC, Mϕ, LC	Defense against fungi and mycobacteria; house dust mite‐induced allergy
DNGR‐1	Dendritic cell natural killer lectin group receptor‐1	Clec9a (Chr. 6, Dectin‐1 cluster)	Hemi‐ITAM	Necrotic cells, mycobacteria	DC, Mo	Necrotic cell Ag cross presentation; defense against *Mycobacterium*
LANGERIN	CD207	Cd207 (Chr. 6)	Proline‐rich motif	Mannose, fucose, β‐glucan	LC	Formation of Birbeck granules; Ag cross‐presentation; antifungal defense
LOX‐1	Lectin‐like oxidized low‐density lipoprotein receptor‐1, CLEC8A, OLR1, HLOX‐1	Clec8a (Chr. 6, Dectin‐1 cluster)	No known motif	Oxidized low‐density lipoprotein	Endothelial cells, Mo, platelets, cardiomyo‐cytes	Progression of atherosclerosis; tumorigenesis
MCL	Mϕ C‐type lectin, CLECSF8, DECTIN‐3	Clec4d (Chr. 6, Dectin‐2 cluster)	None, association with ITAM‐containing FcRg	TDM, Glucurono‐xylomannan	Neu, Mo, Mϕ	Defense against *Mycobacterium* and *Cryptococcus*
MDL‐1	Myeloid DAP12‐assciating lectin‐1	Clec5a (Chr. 6)	None, association with ITAM‐containing DAP12	Dengue virus particle	Mo, Mϕ, osteoclast, Neu	Dengue virus receptor; involvement in inflammation, osteoclastogenesis, arthritis and atherosclerosis; promotion of Mϕ survival
MGL1	Mϕ galactose‐type C‐type lectin‐1, Mϕ asialoglycoprotein‐binding protein 1, MGL, CD301a	Clec10a (Chr. 11)	Hemi‐ITAM (YxxL motif)	Terminal Gal and GalNAc, MUC1, Siglec‐1	Immature DC, Mϕ	Regulation of effector T cell signaling; Ag presentation; suppression of Treg; tumor progression; enhancement of TNF and IL‐10 production
MICL	Myeloid inhibitory C‐type lectin‐like receptor, CLL‐1, DCAL‐2, CD371	Clec12a (Chr. 6, Dectin‐1 cluster)	ITIM	Uric acid crystals	DC, Neu, eosinophils, Mo	Recognition of apoptotic cells; leukemia cancer stem cell marker; Ag uptake and cross‐presentation
MINCLE	Mϕ inducible C‐type lectin, CLECSF9	Clec4e (Chr. 6, Dectin‐2 cluster)	None, association with ITAM‐containing FcRg	TDM, SAP130	Mϕ, DC, Neu, B cells	Defense against fungi and mycobacteria; recognition of damaged cells
MR	Mannose receptor, mannose receptor C‐type 1, MRC1, Mϕ mannose receptor, MMR, CD206	Mrc1 (Chr. 2)	No known motif	Man, Fuc, GlcNAc, lysosomal enzymes, tPA, Gal‐3‐SO4, GalNAc‐4‐SO4, lutropin, CD45, sialoadhesin, MUCIII, *M. tuberculosis* ManLAM	DC, LC, Mϕ, Mo, endothelial cells	Activation of Th2 differentiation and suppression of Th1 differentiation; induction of cytokines in collaboration with TLR2 or DECTIN‐1
SIGN‐R3	Mouse homologue Dendritic cell‐specific intercellular adhesion molecule‐3‐grabbing non‐ integrin (DC‐SIGN), CD209, CD209d, (DC‐SIGN in humans)	Cd209d (Chr. 8) (DC‐SIGN: Chr, 19 in humans)	Hemi‐ITAM (YSDI motif)	Terminal Man and Fuc, Lewis^x^, ManLAM, Lipomannan,LDNF, HIV‐I gp120, ICAM‐2, ‐3	DC, Mϕ	Pathogen recognition; Ag uptake; DC migration; T cell interaction

DC, dendritic cells; LC, Langerhans cells; Mo, monocytes; Mϕ, macrophages; Neu, neutrophils

Many CLRs encoded in the *Dectin‐1* and *Dectin‐2* cluster loci contain ITAM or ITIM in the cytoplasmic domain, suggesting that they transduce signals that regulate cellular function. Some molecules, such as DCAR2, DECTIN‐2, MCL, and MINCLE, have no ITAM but form a complex with ITAM‐containing FcRγ or DAP10/12 to transduce signals. Upon activation of ITAM‐containing CLRs, the SYK kinase is recruited to ITAM and activates CARD9‐BCL10‐MALT1 complex, leading to downstream activation of NF‐κB activation.^2^, ^3^ NF‐κB activation induces various inflammatory cytokines and chemokines, including IL‐1β and IL‐23, which promote Th17 cell differentiation and IL‐17A and IL‐17F production from γδ T cells and type 3 innate lymphoid cells (ILC3s). On the other hand, ITIM‐containing CLRs, including DCIR, recruit a tyrosine phosphatase such as SHP‐1 or SHP‐2 to ITIM and inhibit tyrosine phosphorylation, thereby blocking signals induced by PRRs and cytokines.^4^, ^5^, ^6^ However, DCIR also transduces a positive signal to sustain type 1‐IFNR–induced STAT1 activation in DCs.^7^ By contrast, CLEC1A and LOX‐1 contain no known signaling motifs.

Many ITAM‐containing CLRs encoded in the *Dectin‐1* and *Dectin‐2* cluster loci are thought to play important roles in the host defense against pathogens (Table 1, and see reviews 2 and 3). Upon fungal infection, DECTIN‐1 recognizes β‐(1, 3)‐glucans on the cell wall and activates SYK through ITAM phosphorylation.^8^ DECTIN‐2 recognizes α‐mannans, another PAMP of fungi, and also activates SYK by recruiting FcRγ.^9^ Bacterial components, such as trehalose 6, 6′‐dimycolate (TDM) in mycobacteria, can activate MCL.^10^ SYK activation induces reactive oxygen species (ROS) production, contributing to the eradication of fungi and bacteria. Furthermore, SYK‐induced cytokines, such as IL‐23 and IL‐1β, induce differentiation and production of IL‐17A and IL‐17F from Th17 cells, γδ T cells, and ILC3s. Th17 cytokines play important roles in the eradication of fungi and bacteria by recruiting neutrophils and inducing production of antimicrobial proteins.^11^, ^12^, ^13^, ^14^

Myeloid CLRs have attracted researchers’ attention because recent studies have suggested that they play crucial roles in maintaining immune homeostasis and controlling tumor development, as well as protecting against infection. Because myeloid CLRs can recognize both pathogens and commensal bacteria and fungi, they are important for maintaining the commensal microflora of the skin and the mucoepithelial surface of the intestine and lungs. Dysregulation of these microbiota can cause diseases. Furthermore, some myeloid CLRs recognize endogenous molecules to regulate cell differentiation,^4^, ^15^ and have been suggested to play important roles in the development of inflammatory diseases and tumors by recognizing molecules released by dead cells^16^, ^17^ or expressed on tumor cells.^18^ Hence, in this review, we will discuss the roles of myeloid CLRs in the development of diseases of the skin and mucoepithelial tissues as well as in the development of tumors.

## 2 MYELOID CLRS IN INTESTINAL MUCOSAL IMMUNITY

### 2.1 Colitis

To maintain mucosal homeostasis, the intestinal immune system has to deal with contradictory requirements; the system must be tolerant of commensal microbiota and food components, but fight against invading pathogens. Innate immune receptors such as CLRs and TLRs sense PAMPs not only on pathogens but also on commensal microbiota, and induce ROS, antimicrobial proteins, cytokines, and chemokines that play important roles in the eradication of these pathogens. At the same time, excess cytokines and chemokines cause inflammatory bowel diseases (IBD), including Crohn's disease and ulcerative colitis. Thus, a fine balance between inflammatory (anti‐pathogen) and anti‐inflammatory (tolerogenic) immunity is required for homeostasis of intestinal immunity. However, the mechanisms regulating this balance remain largely obscure.

DECTIN‐1 (gene symbol: *Clec7a*) is the receptor for β‐glucans, which are main components of the fungal cell wall and abundant in the daily diet. Interestingly, unlike other CLRs such as DECTIN‐2 and DCIR, DECTIN‐1 is highly expressed in Mϕs and monocytes of the intestinal lamina propria. Accordigly, this molecule is thought to serve some functions related to intestinal mucosal immunity. Indeed, loss of DECTIN‐1 impairs *Candida albicans*‐specific CD4^+^ T cell development in gastrointestinal‐associated lymphoid tissues,^19^ although DECTIN‐1 is not crucial for defense against intestinal *Candida albicans* infection.^20^ Some pathogenic fungi, such as *Candida tropicalis*, expand in *Clec7a* ^–/–^ mouse intestine and exacerbate the development of dextran sulfate sodium (DSS)–induced colitis.^21^ In the absence of fungal colonization, however, *Clec7a* ^–/–^ mice develop much milder intestinal inflammation than wild‐type mice after DSS administration.^22^ This is because the regulatory T (Treg) cell population is expanded in *Clec7a* ^–/–^ colon due to proliferation of a Treg‐inducing commensal bacterium, *Lactobacillus murinus*. *Lactobacillus murinus* proliferates in *Clec7a* ^–/–^ mice, because levels of the antimicrobial protein calprotectin S100A8, which is induced downstream of DECTIN‐1 signaling through induction of IL‐17F and can inhibit *Lactobacillus* growth, is reduced in these mutant mice.^22^, ^23^ Therefore, DECTIN‐1 acts as a double‐edged sword in the regulation of colitis development; it is necessary for protection against fungal infection, but excess DECTIN‐1 signaling suppresses Treg cell differentiation and induces inflammation. Thus, fungal infection may cause inflammation not only via direct pathogenic effects but also by reducing the abundance of Treg cells in the intestine. Administration of short chain β‐glucans such as laminarin, a component of the brown algae kombu that antagonizes binding of fungal long chain β‐glucans to DECTIN‐1, can ameliorate DSS–induced colitis by increasing the population of Treg cells (20).

In addition, DECTIN‐1 can form a receptor complex with GALECTIN‐3 and FcγRIIB to recognize the mucin MUC2, enhancing oral tolerance by inhibiting NF‐κB activation and inflammatory cytokine production in intestinal DCs.^24^ GALECTIN‐3 promotes the assembly by recognizing N‐glycan structures of not only DECTIN‐1, but also DECTIN‐2 and SIGN‐R1.^25^

Deficiency of CARD9, a downstream adaptor protein of ITAM‐mediated CLR signaling, also causes intestinal fungal expansion and aggravates colitis; oral inoculation of *Lactobacillus murinus* can ameliorate this intestinal inflammation.^26^ The authors of that study suggested that reduced levels of IL‐22, which is important for the recovery from colitis, is responsible for the increased susceptibility to colitis rather than increased fungal growth in *Card9* ^–/–^ mice, and showed that IL‐22 is induced by aryl hydrocarbon receptor ligands produced by commensal bacteria including *Lactobacillus* family members.^26^

**Figure 1:**
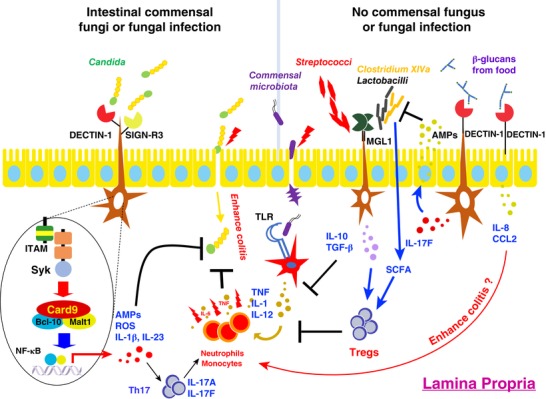
**The roles of CLRs in intestinal mucosal homeostasis**. When the intestinal epithelial barrier is damaged by pathogen infection or physical or chemical injury, microbiota in the intestinal lumen invade the lamina propria, causing inflammation. CLRs are important for the protection against fungal and bacterial infection. Upon *Candida* infection, DECTIN‐1 induces reactive oxygen species (ROS), antimicrobial protein/peptides (AMPs), and Th17 cell differentiation through activation of the SYK‐CARD9‐NF‐κB pathway in order to eradicate the pathogen. SIGN‐R3 expressed on myeloid‐derived cells binds intestinal fungi by recognizing fungal glycan structures. DECTIN‐1 expressed on myeloid‐derived cells also recognizes food‐derived β‐glucans to induce IL‐17F, which stimulates epithelial cells to secret antimicrobial protein S100A8 and phospholipase A2 to inhibit the growth of *Lactobacillus murinus* and *Clostridium* cluster XIVa, respectively. Both *Lactobacillus* and *Streptococcus* are recognized by MGL1 and induce IL‐10 and TGF‐β, promoting the expansion of Treg cells and also directly suppressing inflammation. *Clostridium* XIVa also induces Treg differentiation by producing short‐chain fatty acids (SCFA). On the other hand, DECTIN‐1 expressed on human intestinal epithelium induces the production of IL‐8 and CCL2 after β‐glucan stimulation, which may recruit neutrophils to mediate inflammation

DECTIN‐1 is expressed in freshly isolated human intestinal epithelial cells (IECs) and human IEC lines, but not in the analogous mouse cells.^27^ Stimulation of human IECs with β‐glucans induces IL‐8 and CCL2 secretion, which can be blocked by SYK inhibition,^27^ suggesting involvement of IECs in the development of colitis in humans. *CLEC7A* expression is up‐regulated in inflamed colons of IBD patients,^28^ and 2 single‐nucleotide polymorphisms in *CLEC7A* are correlated with medically refractory ulcerative colitis.^21^ Thus, DECTIN‐1 plays important roles not only in host defense against infection, but also in maintaining intestinal homeostasis under physiological conditions (Fig. 1). However, additional studies are still needed to fully understand the roles of this molecule completely in the homeostasis of human intestinal immunity.

Other myeloid CLRs are also implicated in the development of colitis. MGL1, expressed in colonic lamina propria F4/80‐high cells, binds *Streptococcus* species and *Lactobacillus* species to induce IL‐10 production in vitro.^29^ Mice lacking this molecule develop more severe inflammation after DSS‐treatment, accompanied with impaired IL‐10 secretion.^29^ Although MCL and DCIR also bind some intestinal commensal microbiota, mice deficient in these molecules develop slightly more severe DSS–induced colitis.^30^ Another report showed that DCIR‐deficient mice develop even milder colitis, with reduced neutrophil‐attracting chemokine MIP‐2 and decreased accumulation of neutrophils.^31^ MR‐expressing mouse intestinal Mϕs contribute to wound healing in DSS‐induced colitis.^32^ SIGN‐R1, the mouse homolog of human DC‐SIGN, synergizes with TLR4 to respond to LPS, and deficiency of SIGN‐R1 impairs commensal bacteria‐induced pro‐inflammatory cytokine production and attenuates intestinal inflammation after DSS administration.^33^ Another DC‐SIGN homolog, SIGN‐R3, also recognizes glycan structures on commensal fungi and *Mycobacterium tuberculosis*. Mice deficient in this molecule are more sensitive to *M. tuberculosis* infection and develop more severe colitis with an enhanced TNF production.^34^ The detailed functional roles of these myeloid CLRs remain to be elucidated.

## 3 MYELOID CLRS IN PULMONARY MUCOSAL IMMUNITY

### 3.1 Asthma and allergic diseases

**Figure 2:**
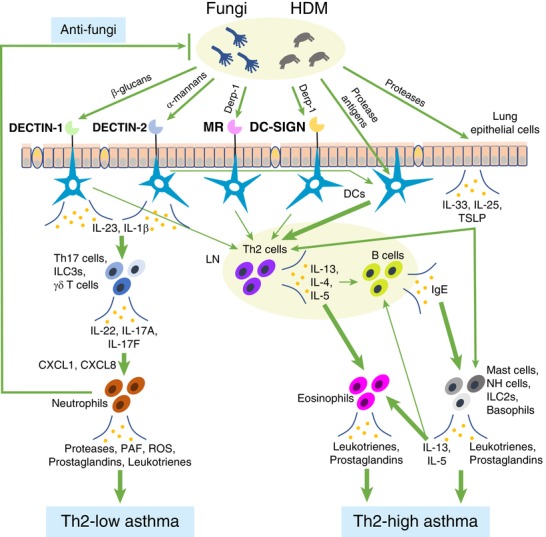
**The roles of CLRs in asthma**. Ags from *Aspergillus fumigatus* and HDM activate DCs to induce differentiation of Th2 cells, resulting in the development of Th2‐high asthma through activation of mast cells, eosinophils, NH cells, ILC2s, and basophils. On the other hand, PAMPs from these microbes activate CLRs such as DECTIN‐1 and DECTIN‐2, leading to the differentiation of Th17 cells and activation of γδ T cells and ILC3s. IL‐17A and IL‐17F produced in these cells induce inflammation in the lungs by recruiting neutrophils, that is a characteristic of Th2‐low, steroid resistant asthma. IL‐22 produced in these cells, as well as IL‐17A and IL‐17F, is involved in mucus production and epithelial proliferation. DECTIN‐1, DECTIN‐2, MR, and DC‐SIGN also take part in the activation of Th2 cells, facilitating the development of Th2‐high, steroid‐sensitive asthma

Asthma, one of most common chronic respiratory diseases, is associated with airway inflammation and remodeling. Possible alterations of asthmatic patient airway structure include mucous gland and goblet cell hyperplasia, modification of epithelial cells, subepithelial fibrosis, constriction of airway smooth muscle, and changes in blood vessels.^35^ Numerous cytokines, including IL‐9, IL‐13, IL‐17, IL‐22, IL‐25, and other inflammatory mediators, are involved in the airway remodeling.^36^, ^37^ Despite being classified as a single disease, the term “asthma” subsumes pathologically distinct complex diseases, often accompanied by other morbidities, complicating patient state and decisions about treatment regimen.^38^ Frequently, asthma originating in childhood may continue at older ages. To a large extent, asthma developed in children is associated with allergy and atopic disease. Atopic asthma is caused by type 2 immune responses with enhanced IgE production, followed by eosinophilia and mast cell activation.^39^ Asthma can also be diagnosed at any age in adulthood. However, the majority of adult‐diagnosed asthma is Th2‐low and non‐atopic, and is often associated with high neutrophil concentrations and elevated Th17‐related responses.^40^, ^41^ Importantly, Th2‐high and Th2‐low forms of asthma exhibit distinct responses to corticosteroid treatment; Th2‐high asthmatics respond to this treatment, whereas Th2‐low patients are refractory.^38^ CLRs are thought to be involved in both forms of asthma (Fig. 2).

#### 3.1.1 *Aspergillus fumigatus*‐associated asthma

Sensitization to allergens from *Aspergillus fumigatus* is often associated with asthma. A study by Bozza et al. revealed that various fungal components are responsible for different Th‐type responses and cytokine production.^42^ Secreted proteins such as metalloprotease (Mep1p), superoxide dismutase (Sod1p), and ribonuclease (RNUp) from the fungus induce Ag‐specific Th2‐cell differentiation. IL‐5 and IL‐13 from Th2 cells promote IgE production from B cells, which leads to activation of mast cells and basophils by the immune complex to produce various inflammatory mediators such as leukotrienes and prostaglandins. Th2 cells also recruit eosinophils and mast cells and promote production of these inflammatory mediators. Furthermore, fungal and house dust mite (HDM) proteases and a fungal glycosphingolipid release IL‐33 from epithelial cells and Mϕs, respectively.^43^, ^44^ IL‐33 directly induces the production of inflammatory mediators from basophils, mast cells, and eosinophils, and also indirectly induces inflammatory signaling by promoting Th2 cytokine production from Th2 cells, ILC2, mast cells, and basophils, mostly resulting in development of steroid‐sensitive asthma,^42^, ^45^, ^46^, ^47^ although some reports have suggested that this phenomenon is involved in steroid resistant asthma^48^ (Fig. 2).

Both DECTIN‐1 and DECTN‐2 are predominantly involved in the antifungal response, and their expression levels increase upon infection with the hyphal form of *Aspergillus fumigatus*.^8^, ^9^, ^49^, ^50^ Interestingly, polysaccharides from the fungal cell wall, namely, β‐1,3‐glucans and α‐mannans, can induce Th17 cell differentiation by inducing cytokines, including IL‐1β in DCs through activation of DECTIN‐1 and IL‐23 in Mϕs through activation of DECTIN‐2.^9^, ^51^, ^52^ Th17 cell‐derived IL‐17A and IL‐17F recruit neutrophils to eradicate fungi, and at the same time, induce steroid‐resistant lung inflammation.^53^ Upon infection with *Aspergillus fumigatus*, neutrophils are activated in an autocrine manner by expressing IL‐17A and IL‐17RC via DECTIN‐2‐mediated induction of IL‐6 and IL‐23.^49^, ^54^ DECTIN‐2 is also involved in Th2‐type asthma in response to both *Aspergillus fumigatus* and HDM.^55^, ^56^ Barrett et al. revealed that DECTIN‐2 can stimulate production of cysteinyl leukotrienes by DCs in response to *Aspergillus fumigatus* extract.^56^ These leukotrienes produced by DCs are potent mediators of pulmonary inflammation in bronchial asthma and can augment Th2 sensitization.^55^, ^56^ DECTIN‐1 is also a potent inducer of leukotrienes in mast cells and Mϕs after stimulation with zymosan.^26^, ^27^ Furthermore, DECTIN‐1 induces IL‐22, which aggravates airway hypersensitivity (AHR) by promoting the production of proallergic chemokines and mucus, along with IL‐17A and IL‐17F.^57^ β‐Glucans from *Aspergillus versicolor*, a close relative of *Aspergillus fumigatus*, worsens HDM–induced AHR by causing a mixed inflammatory reaction involving both Th2 and Th17 cells, accompanied by increased number of neutrophils and eosinophils.^58^ Similar enhancement of asthma is observed when mice are treated with a combination of β‐glucans and LPS.^59^ Although acute exposure of *Clec7a* ^–/–^ mice to *Aspergillus fumigatus* increases fungal invasion of the fungus, it induces milder allergic response with reduced neutrophil infiltration.^57^ Thus, DECTIN‐1 (and possibly DECTIN‐2) are primarily involved in the induction of Th17 responses in asthma accompanied by high neutrophilic infiltration, although direct evidence for the involvement of DECTIN‐2 in asthmatic Th17 responses is lacking. On the other hand, the asthmatic response of allergic bronchopulmonary aspergillosis (ABPA) patients to *Aspergillus fumigatus* mainly is mediated not by DECTIN‐1‐induced neutrophilia but by allergic Th2‐type responses induced by fungal proteases.^60^ In addition, *Aspergillus fumigatus*‐derived proteases and neutrophil elastase can cleave DECTIN‐1, DECTIN‐2, and MINCLE, suppressing antifungal immune responses and promoting development of ABPA in cystic fibrosis patients.^61^

Among other CLRs, DC‐SIGN, the receptor for galactomannans, is also suggested to take part in *Aspergillus fumigatus*‐induced immune responses by recognizing galactomannans on *Aspergillus fumigatus*.^54^, ^62^ However, its role in allergy‐related processes remains to be elucidated.

#### 3.1.2 HDM‐associated asthma

HDMs are another widespread cause of AHR response and allergy.^63^ HDM allergens include proteases of *Dermatophagoides pteronyssinus* (Der p 1, Der p 3, Der p 6, and Der p 9) and of *Dermatophagides farina* (Der f 1 and so on), which can induce production of inflammatory cytokines, breakage of epithelial barriers, and stimulation of airway smooth muscle proliferation in asthmatic patients.^63^ Some components of HDMs, such as chitin and β‐glucans, are thought to act as PAMPs, resulting in activation of immune responses via several pathways including CLRs.^64^ DECTIN‐1 on CD11b^+^ DCs binds to components of HDM extracts and modulates both Th2‐ and Th17‐related immune responses; the production of IL‐5, IL‐13, and IL‐17A, as well as chemokines CCR7, CCL3, and CCL4, is reduced in *Clec7a* ^–/–^ mice upon HDM exposure,^65^ although the ligands for DECTIN‐1 remain to be identified. Data concerning the role of chitin in allergic responses are very limited. Da Silva et al. showed that mammalian chitinase cuts originally intact molecules into pieces. Depending on their sizes, these pieces are recognized by DECTIN‐1 in collaboration with TLR2, resulting in production of TNF, or by DECTIN‐1 and MR, resulting in production of IL‐10.^66^ FIBCD1 has been reported as a receptor for chitin, but its role in asthma remains to be elucidated.^67^

Components of HDM extract also trigger cysteinyl leukotriene generation by CD11c^+^ DCs through the activation of the DECTIN‐2–SYK pathway,^56^ and activates Th2 immune responses.^55^ Moreover, DECTIN‐2–blocking Abs ameliorate Th2 inflammation through the attenuation of inflammatory cytokines such as IL‐4, IL‐5, and IL‐13, and chemokines CCL22 and CCL17.^68^, ^69^ *Clec4n* ^–/–^ mice also show significantly attenuated HDM‐induced allergic airway inflammation and less extensive Th2 and Th17 cell differentiation associated with reduced levels of inflammatory cytokines and chemokines.^68^, ^70^ Thus, HDM‐activation of DECTIN‐2 accelerates airway allergic responses by inducing Th2 and Th17 cytokines and chemokines, although the DECTIN‐2 ligands on HDMs have not yet been identified.

The role of MR and DC‐SIGN in the HDM‐related response was previously reviewed by Hadebe et al.^64^, ^71^ CD206 and DC‐SIGN are receptors for HDM allergens (Der p 1 and Der p 2). CD206 induces Th2 polarization after stimulation with Der p 1 by up‐regulating indoleamine 2,3‐dioxigenase activity.^71^ DC‐SIGN also promotes Th2 cell polarization upon interaction with Der p 1, because the protease cleaves DC‐SIGN, which is in turn important for Th1 differentiation.^72^

These observations suggest that CLRs play versatile roles in the development of asthma. CLRs such as DECTIN‐1 and DECTIN‐2 are primarily important for the defense against allergic fungal infection, but they also promote asthma pathogenesis by promoting Ag‐specific allergic responses. In particular, these CLRs make important contributions to the development of Th2‐low, steroid‐resistant asthma by promoting Th17 immune responses. Thus, suppression of CLRs should be beneficial for the treatment of asthma, but caution is necessary because this strategy may also promote fungal growth.

## 4 MYELOID CLRS IN CUTANEOUS IMMUNITY

### 4.1 Psoriasis

**Figure 3:**
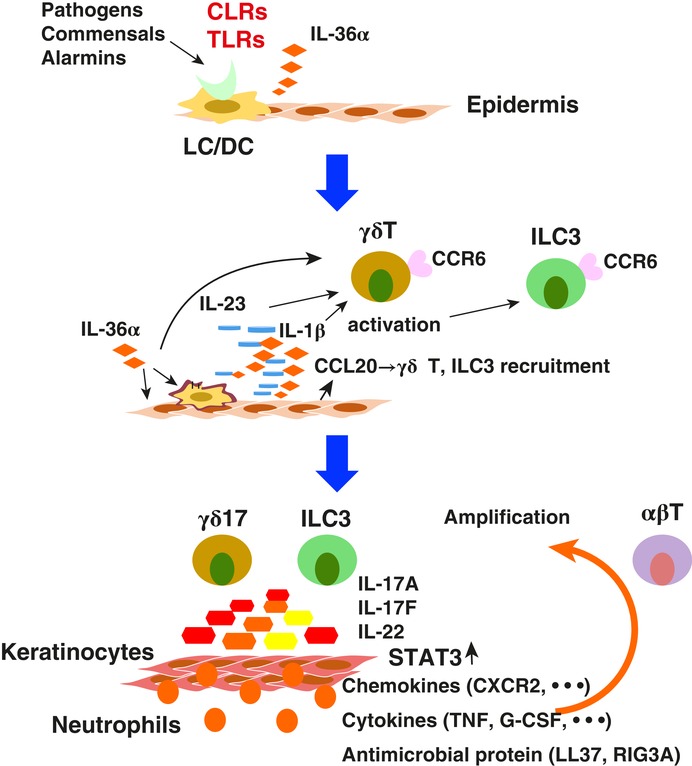
**The roles of CLRs in psoriasis**. PAMPs from pathogens or commensal microbiota or alarmins from dead skin cells activate CLRs and TLRs such as DECTIN‐1 or TLR7 on Langerhans cells (LCs), leading to production of proinflammatory cytokines including IL‐36α, IL‐23, and IL‐1β. IL‐36α also induces IL‐23, IL‐1β, and CCL20 in LCs and keratinocytes. Then, CCL20 recruits γδ T cells and ILC3 to the inflammatory sites, and IL‐23 and IL‐1β activate these cells to produce IL‐17A, IL‐17F, and IL‐22. These cytokines recruit neutrophils and activate keratinocytes to produce various inflammatory cytokines, chemokines, and antimicrobial peptides such as TNF, G‐CSF, CXCR2, LL37, and REG3A, causing development of inflammation and keratinocyte proliferation. These chemokines and cytokines further activate not only γδ T cells and ILC3 but also αβ T cells to enhance these inflammatory processes, forming an amplification loop

Psoriasis is a chronic inflammatory skin disease characterized by thickening and redness of the skin associated with keratinocyte hyperproliferation, skin inflammation with inflammatory cell infiltration in the epidermis and dermis, and (in severe cases) aseptic abscess formation.^73^ Cytokines such as IL‐23, IL‐17A, and TNF play important roles for the pathogenesis of the disease, and the Abs against these cytokines are effective in treating the disease.^74^, ^75^ Innate immune responses play important roles in mouse models. Imiquimod (IMQ), a TLR7 ligand, induces psoriasiform dermatitis in mice by inducing IL‐23^76^ from Langerin‐negative conventional DCs (cDCs),^77^ followed by downstream induction of IL‐17A. IL‐17A is mainly produced by γδ T cells and ILC3, but not by αβ T cells.^78^ IL‐17F is also involved in the development of dermatitis.^78^ Interestingly, IL‐36α may be important for the induction of IL‐23.^79^ IMQ directly induces IL‐36α in bone marrow‐derived Langerhans cells and GM‐CSF‐induced DCs, and IL‐36α acts on bone marrow‐derived Langerhans cellss and keratinocytes to produce IL‐23, IL‐1β, and chemokines such as CCL20, CXCL1, and CXCL2,^80^ which recuit γδ T cells and ILC3 and induce IL‐17A in these cells.^81^ Thus, IL‐23 is induced directly in cDCs by IMQ, and indirectly in LCs and keratinocytes through induction of IL‐36α. Because IL‐36α is induced not only by IMQ but also by β‐glucans from *Candida albicans*, fungal infection may also be involved in the development of dermatitis.^80^ It is likely that various innate immune receptors such as TLRs and CLRs expressed on the cell surface of LCs and DCs of the skin recognize PAMPs of bacteria and fungi, as well as alarmins derived from dying cells, and induce cytokines and chemokines including IL‐36α, IL‐23, IL‐1β, and CCL20. Then, CCL20 recruits γδ T cells and ILC3 to inflammatory sites, and IL‐23 and IL‐1β activate these cells to produce IL‐17A, IL‐17F, and IL‐22. This leads to recruitment of neutrophils and production of various inflammatory cytokines and chemokines including TNF, G‐CSF, IL‐1β, CXCL1, and CXCL2 from keratinocytes and fibroblasts, causing inflammation and promoting keratinocyte proliferation, ultimately resulting in hyperplasia of the skin (Fig. 3). The antimicrobial protein LL37, which is produced by keratinocytes, is thought to activate TLR7 and TLR8 by forming a complex with RNA.^82^ Thus, innate immune responses, but not acquired immune responses, may play central roles in the development of psoriasis. After initiation of inflammation, however, various inflammatory cytokines may also activate immune cells of the acquired immune system, enhancing the inflammatory processes by forming an amplification loop.

Involvement of CLRs is also suggested in the development of psoriasiform dermatitis and arthritis in SKG mice. Administration of β‐glucans or mannans evokes these symptoms in these mice, which do not develop any symptoms under SPF conditions, and the development of arthritis is suppressed in *Clec7a* ^–/–^ mice, suggesting that innate immunity triggers the development of Ag‐dependent autoimmunity.^83^, ^84^ Innate immune signaling has been suggested to activate complement pathways and produce C5a, which stimulates Mϕs to produce IL‐6 and GM‐CSF, which in turn promote Th17 differentiation.^85^ Induction of IL‐17A in Th17 cells is strictly TCR dependent, in contrast to the situation in γδ T cells or ILC3s. Treatment of psoriatic patients with corticosteroids or biologics targeting activated T cells or costimulation of T cells is clinically effective,^82^ suggesting that Th17 cells other than γδ T cells and ILC3s may also be involved in the development of psoriasis.

A Mϕ mannose receptor (MR) is expressed on immature DCs, but not on mature DCs or Langerhans cells. MR^+^ inflammatory dendritic epidermal cells are present in samples of skin from patients with atopic dermatitis (AD) or psoriasis, and use this receptor for receptor–mediated endocytosis of mannans.^86^ MR is suggested to regulate the development of psoriasiform dermatitis in mannan–injected mice, because MR‐deficient mice develop more severe mannan–induced dermatitis, associated with the reduced production of ROS, which is important for the differentiation of immunosuppressive M2 Mϕs.^87^ On the other hand, MR is expressed in CD163^+^ dermal Mϕs together with DC‐SIGN. CD163^+^ Mϕs produce IL‐23 as well as TNF and inducible nitric oxide synthase, suggesting that these Mϕs play pathogenic roles in psoriasis.^88^ Thus, further work is needed to elucidate the roles of MR in the development of psoriasis.

Although the expression of other myeloid CLRs, such as Langerin and DC‐SIGN, is elevated in DCs and keratinocytes in psoriatic skin, the functional roles of these CLRs still remain obscure.^77^, ^89^ Elevated expression of these molecules may reflect accumulation and/or activation of Langerhans cells and DCs and altered differentiation of keratinocytes.

### 4.2 Atopic dermatitis

AD is a chronic inflammatory skin disease associated with intense itch and recurrent eczematous lesions. The pathophysiology of AD is complex and multifactorial, and barrier dysfunctions of the skin such as caused by mutations in *FILAGGRIN* and enhanced cell‐ and IgE‐mediated immune responses caused by sustained infection of bacteria and fungi are thought to be critically involved in the pathogenesis.^90^, ^91^

AD patients have an elevated susceptibility to infection with bacteria, fungi, and viruses,^90^, ^91^ and CLRs play important roles in the protection of pathogen invasion and pathogenesis of dermatitis. Among these pathogens, the best characterized is *Staphylococcus aureus*, which is detected in approximately 90% patients and is associated with disease exacerbation. The non‐myeloid CTL mannose‐binding lectin contributes to defense against this bacterium by activating the complement lectin pathway via an interaction with specific polysaccharide structures. *Malassezia*, a commensal fungus on the skin, is also thought to cause AD by producing a variety of immunogenic proteins that elicit specific IgE immune responses.^92^, ^93^ MINCLE, expressed on activated phagocytes, can recognize *α‐mannosyl* residues on *Malassezia*, resulting in the activation of Mϕs to produce inflammatory cytokines and chemokines.^94^ Mast cells from AD patients also express MINCLE, and upon exposure to *Malassezia, Mincle* expression, and IL‐6 secretion are enhanced.^95^

DECTIN‐1 expression is higher in AD skin compared to healthy skin.^6^ However, stimulation of *Dectin‐1* expression by *Malassezia* or IgE crosslinking is impaired in AD‐derived mast cells,^6^ suggesting a defect in defense against fungal infection in AD patients. DECTIN‐1 signaling suppresses Th2 immune responses induced by epicutaneous OVA sensitization associated with reduction of IL‐4 and IL‐1β expression.^96^

DC‐SIGN expression on DCs is high in the lesional skin of AD patients, and the level is associated with disease severity. Zhang et al. suggested that DC‐SIGN on DCs binds common allergens such as HDM allergen (Der p 2) and egg white allergen (Gal d2) and initiates allergen sensitization or provokes AD relapse by inducing proinflammatory cytokines including TNF and IL‐6 to facilitate Th2 and Th22 polarization.^97^ On the other hand, Smits et al. showed that DC‐SIGN binds *Lactobacillus reuteri* and *Lactobacillus casei*, but not *Lactobacillus plantarum*, driving the differentiation of Treg cells by stimulating monocyte‐derived DCs,^98^ suggesting that targeting of DC‐SIGN by certain probiotic bacteria may be beneficial to treat AD.

## 5 MYELOID CLRS IN TUMOR IMMUNITY

**Figure 4:**
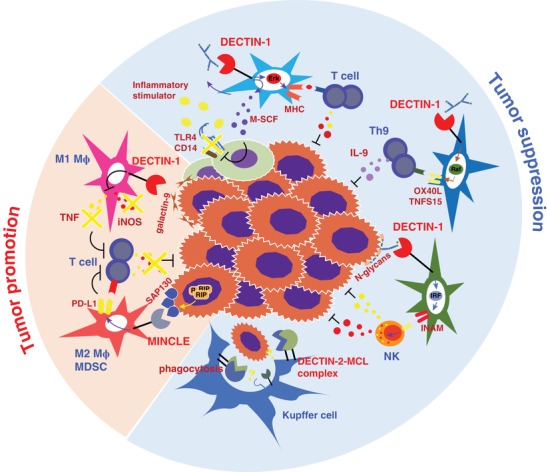
**The roles of CLRs in tumor immunity**. By suppressing TLR4 and CD14 expression, DECTIN‐1 can inhibit inflammation–induced hepatocellular carcinogenesis. β‐Glucan stimulation enhances MHC‐II^+^ anti‐tumor myeloid–derived cell differentiation through Erk activation to suppress lung carcinoma. DECTIN‐1 also activates Raf1 to express TNFSF15 and OX40L to promote anti‐tumorigenic Th9 differentiation. Furthermore, by binding to glycoprotein N‐glycans on B16 melanoma cells, DECTIN‐1 enhances tumor‐killing activity of NK cells through IRF5‐dependent INAM induction. DECTIN‐2 and MCL expressed on liver resident Kupffer cells increase the antitumor phagocytotic activity of these cells. By contrast, DECTIN‐1 recognizes noncanonical endogenous ligand galectin‐9 expressed on pancreatic cancer cells and suppresses M1 Mϕ‐mediated T cell antitumor immunity. In pancreatic ductal adenocarcinoma, MINCLE recognizes the cytoplasmic histone deacetylase complex SAP130 to promote MDSC‐mediated immune suppression, thereby down‐regulating antitumor immunity

Antitumor immunity is important for protection and eradication of tumors. CLRs are members of the immune surveillance system and are thought to recognize tumor‐specific Ags or neo‐Ags to activate antitumor immunity. Both acquired immune cells, especially cytotoxic CD8^+^ T cells (CTLs), and innate immune cells such as NK cells play important roles in the eradication of tumor cells.^99^, ^100^ On the other hand, Treg cells and myeloid‐derived suppressor cells (MDSCs) interfere with the antitumor immunity.^101^ PD‐L1 and/or PD‐L2 expressed on some tumor cells also inhibit antitumor immunity by interacting with PD‐1 on cytotoxic T cells.^101^ In the course of tumor development, cancer cells frequently metastasize and relocate to other organs through nearby blood vessels. Although CLRs are thought to be involved in these complicated immune processes, their functional roles have not been fully elucidated (Fig. 4).

DECTIN‐1 is suggested to play a protective role by directly recognizing tumor‐specific Ags. By binding to glycoprotein N‐glycans on B16F1 melanoma cells, DECTIN‐1 enhances tumor‐killing activity of NK cells through induction of INAM and other molecules on DCs and Mϕs in an IRF5–dependent manner.^18^ Furthermore, DECTIN‐1 activates Raf1 and NF‐κB to express TNFSF15 and OX40L on DCs to promote the differentiation of antitumorigenic Th9 cells.^102^ DECTIN‐1‐induced IL‐33 also contributes to the induction of Th9 cells.^103^ DECTIN‐1 can also suppress liver inflammation induced by chemical carcinogens, which results in fibrosis and hepatocellular carcinogenesis, by suppressing the expression of TLR4 and CD14 through induction of M‐CSF.^104^ Interestingly, oral administration of yeast‐derived β‐glucan particles suppresses the growth of subcutaneously inoculated Lewis lung carcinoma, by inducing polymorphonuclear MDSC apoptosis and monocytic MDSC differentiation to MHC‐II^+^ antitumor APCs through Erk1/2 activation.^105^ On the other hand, DECTIN‐1 expressed on Mϕs in mice and humans recognizes the noncanonical DECTIN‐1 ligand galectin‐9, which is abundantly expressed on pancreatic ductal adenocarcinoma cells, and suppresses M1 Mϕ differentiation and T cell‐mediated antitumor immunity, suggesting a tumor promotive role of DECTIN‐1 signaling in pancreatic tumors.^106^ Furthermore, administration of a DECTIN‐1 antagonist, laminarin, to *A4gnt*–deficient mice, a model for spontaneous gastric adenocarcinoma, suppresses gastric dysplasia and attenuates epithelium angiogenesis.^107^ Therefore, DECTIN‐1 plays opposing roles in tumorigenesis depending on the microenvironments of different types of cancers. The precise conditions controlling these functions should be clarified before treatments targeting DECTIN‐1 are applied in the clinic.

The genes encoding DECTIN‐2, MCL, and MINCLE are mapped in close proximity in the *Dectin‐2* cluster, and MCL can form heterodimers with DECTIN‐2 and MINCLE, implying that these CLRs have related immunological functions even though they recognize distinct ligands. DECTIN‐2 and MCL are expressed not only on lymphoid tissues, but also on alveolar Mϕs and liver‐resident Kupffer cells, which resemble Mϕs. Deficiency of either DECTIN‐2 or MCL leads to exacerbated liver metastasis after intrasplenic inoculation with SL4 colon carcinoma or B16F1/10 melanoma cells, accompanied by impaired phagocytotic activity of Kupffer cells,^108^ suggesting that these CLRs enhance Kupffer cell‐mediated tumor phagocytosis. Similarly, DECTIN‐1 deficiency results in severe metastasis of the melanoma cells. In these mice, however, impaired killing activity of nonparenchymal NK cells is suggested to be responsible for the defect of antitumor activity.^108^ On the other hand, in a pancreatic ductal adenocarcinoma model, MINCLE is up‐regulated in tumor‐infiltrating Mϕs; in addition, by recognizing a subunit of cytoplasmic histone deacetylase complex SAP130, MINCLE promotes oncogenesis by enhancing Mϕ‐induced immune suppression.^109^

Expression of CLRs is correlated with development of some human cancers, but the underlying mechanisms remain unknown. Serum levels of soluble DC‐SIGN are reduced in colon cancer patients, and high serum levels of soluble DC‐SIGN correlate with long‐term survival, suggesting that this molecule could serve as a novel prognostic biomarker.^110^ MICL is detected on acute myeloid leukemia CD34^+^ stem cells, and mAbs against this molecule cause Ab‐dependent cellular cytotoxicity against both cultured and freshly isolated leukemia cells, suggesting a new therapeutic strategy against acute leukemia.^111^

Recently, the SYK‐CARD9 signaling pathway, the common downstream of fungal recognition CLRs such as Dectin‐1 or Dectin‐2, was shown to be involved in anti‐colorectal cancer immunity. Commensal gut fungi activate inflammasomes through the SYK‐CARD9 pathway, resulting in the suppression of AOM‐DSS–induced colitis and colon tumorigenesis by promoting epithelial barrier restitution via enhancement of IL‐18 maturation and IFN‐γ production in CD8^+^ T cells.^112^ On the other hand, development of AOM‐DSS–induced tumors is enhanced in *Card9* ^–/–^ mice, accompanied by an increase in the fungal burden in the intestine, which causes the accumulation of tumor‐promoting MDSCs.^113^ These results suggest that intestinal fungi can either attenuate or promote intestinal tumor development, leaving obscure the exact roles of fungus‐induced CLR signaling in the intestinal tumorigenesis.

## 6 CONCLUDING REMARKS

In this review, we described the roles of myeloid CLRs in diseases of muco‐epithelial tissues. Recent progress in research on myeloid CLRs has revealed that in addition to the host defense against pathogens, these molecules play important roles in the homeostasis of muco‐epithelial immunity and development of diseases, including colitis, asthma, psoriasis, atopic dermatitis, and cancers. The functions of these CLRs are complex, and their roles in diseases, their ligands, and the detailed mechanisms underlying their actions remain largely unknown. Elucidation of the physiological as well as pathological roles of these CLRs may provide us with clues that could aid in the development of new therapeutics against these diseases.

## AUTHORSHIP

C.T. and Y.M. are co‐first authors of the study.

## DISCLOSURE

The authors declare no conflict of interest.
